# Geospatial analysis of cardiovascular mortality before and during the COVID-19 pandemic in Peru: analysis of the national death registry to support emergency management in Peru

**DOI:** 10.3389/fcvm.2024.1316192

**Published:** 2024-06-03

**Authors:** Jeel Moya-Salazar, Eileen A. Marín, Camila B. Palomino-Leyva, Jhonny Rivera, Rosario La Torre, Betsy Cañari, Claudio Pardo-Villarroel, Hans Contreras-Pulache

**Affiliations:** ^1^ Digital Transformation Center, Universidad Privada del Norte, Lima, Peru; ^2^ Faculties of Health Science, School of Medicine, Universidad Científica del Sur, Lima, Peru; ^3^ Neuroscience Unit, Nesh Hubbs, Lima, Peru; ^4^ Department of Environmental Sciences, Faculty of Natural Resources, Universidad Católica de Temuco, Temuco, Chile

**Keywords:** cardiovascular diseases, COVID-19, spatial analysis, myocardial infarction, analytical and synthetic maps, heart failure, Peru

## Abstract

**Background:**

COVID-19 has led to significant global mortality, with Peru being among the countries most affected. While pre-existing comorbidities have been linked to most cases, the exact distribution of fatalities within the country remains unclear. We aimed to assess deaths attributed to cardiovascular diseases (CVD) before and during the COVID-19 pandemic across various regions and provinces in Peru.

**Methods:**

An observational georeferencing study was designed. Peru faced four waves of COVID-19 over three years, with variable impacts across its three regions (Coast, Highlands, and Jungle). Deaths related to cardiovascular diseases, such as heart failure (HF), arrhythmia, acute myocardial infarction (AMI), strokes, and acute coronary syndrome, were examined as primary variables. The study period spanned pre-pandemic years (2017–2019) and pandemic years (2020–2021), utilizing death data from the National Death Information System (SINADEF). The georeferencing analysis was conducted using ArcGIS v10.3.

**Results:**

A total of 28,197 deaths were recorded during the study period, with significant increases during the pandemic (2020–2021). Cardiovascular deaths were disproportionately higher during the pandemic, totaling 19,376 compared to 8,821 in the pre-pandemic period (*p* < 0.001). AMI and HF were the leading causes of mortality, showing significant increases from the pre-pandemic (5,573 and 2,584 deaths) to the pandemic period (12,579 and 5,628 deaths), respectively. Deaths due to CVD predominantly affected individuals aged over 60, with significant increases between the two study periods (7,245 vs. 16,497 deaths, *p* = 0.002). Geospatial analysis revealed regional disparities in CVD mortality, highlighting provinces like Lima and Callao as COVID-19 critical areas. The substantial increase in cardiovascular deaths during the COVID-19 pandemic in Peru showed distinctive patterns across regions and provinces.

**Conclusions:**

Geospatial analysis identified higher-risk areas and can guide specific interventions to mitigate the impact of future health crises. Understanding the dynamic relationship between pandemics and cardiovascular health is crucial for effective public health strategies.

## 1 Introduction

COVID-19 waves caused by SARS-CoV-2 have had significant health implications, particularly for the elderly and individuals bearing global risk factors (1). In Latin America, the impact of the pandemic has been devastating, resulting in high mortality rates in countries like Brazil, Ecuador, and Peru (2). Sempé et al. in 2021 reported a 73.9% excess mortality associated with COVID-19 among Peruvian adults aged over 60 (3), while the National Institute of Health of Peru indicated that the elderly group had the highest mortality rates (4).

Several factors can influence COVID-19 mortality rates, with cardiovascular dis-eases being a significant contributor to the pandemic burden (5). Banerjee et al. observed mortality rate fluctuations related to cardiovascular diseases in England, while a meta-analysis has highlighted the association between mortality rates and cardiovascular disease prevalence in COVID-19 patients, including acute cardiac injury, hypertension, arrhythmia, heart failure (HF), coronary heart disease, and stroke (6, 7).

Even before the onset of the COVID-19 pandemic, cardiovascular diseases were already widespread, displaying elevated incidence and mortality rates (5). Furthermore, existing cerebrovascular and cardiovascular conditions have been linked to heightened susceptibility and severity of SARS-CoV-2 infections, which in turn can worsen preexisting cardiovascular ailments or give rise to new cardiac complications (8). Thus, emphasizing the importance of monitoring and managing cardiovascular diseases in COVID-19 patients is crucial, along with prioritizing preventive measures to mitigate the overall disease burden.

Georeferencing methods have proven useful in identifying potential disease risk areas through geospatial statistics (9). During the COVID-19 pandemic, georeferencing methods have been used to track cases (10), evaluate vaccination efforts (7), and assess unvaccinated children (9). Despite this, deaths related to cardiovascular diseases in Peru have not been regionally monitored, and their overall impact has not been estimated as part of the national chronic disease surveillance.

Therefore, the main objective of our geospatial study was to determine deaths attributed to cardiovascular diseases, both before and during the COVID-19 pandemic, in different regions and provinces of Peru. Furthermore, we aimed to compare mortality rates between the pre-pandemic period (<2019) and the pandemic period (2020–2021) to understand variations in types and frequencies of cardiovascular dis-eases.

## 2 Material and methods

### 2.1 Study design and geographical location

We designed an observational study based on georeferencing in the context of COVID-19 in Peru. Peru, a middle-income country with a population of 33.72 million, is situated in South America (latitude −9.189967) and is divided into three regions (Coast, Highlands, and Jungle) and 24 departments (11). The study area data are presented in Table 1.

**Table 1:** Baseline information of study area.

Items	Characteristics
Location	Central and western region of South America (between the parallels 0°2′ and 18°21'34″ south latitude, and the meridians 68°39'7″ and 81°20'13″ west longitude).
Limits	To the north with Ecuador and Colombia, to the east with Brazil, to the southeast with Bolivia, to the south with Chile, and to the west with the Pacific Ocean.
Surface	1,285 square kilometers of land and 200 nautical miles of sea
Climate	Arid and temperate on the coast, rainy and cold in the highlands, and very rainy and warm in the jungle.

### 2.2 COVID-19 in Peru

The first COVID-19 case in Peru was reported in Lima on March 6, 2020, in a 25-year-old man returning from Europe. Thirteen days later, with 234 reported infected cases nationwide, the first death was recorded (12). Over the course of three years, Peru experienced four COVID-19 waves. The first wave (starting March 16, 2020) introduced quarantine, mobility restrictions, and tracking of suspected cases and isolation (13). In the second wave (starting July 16, 2021), unlike the previous wave with a high seropositivity rate, the second wave was more lethal due to the lambda and SARS-CoV-2 gamma variants (14). On January 5, 2022, the third wave of COVID-19 began, with the omicron variant being predominant in the Lima metropolitan area (15). Finally, on June 27, 2022, the fourth wave of COVID-19 infections began, recording approximately 10,826 new cases (16).

### 2.3 Variables and instruments

The variables of the study included deaths from major cardiovascular diseases such as HF, arrhythmia, heart attack, stroke, and acute coronary syndrome (AMI) (17, 18). Additionally, another variable was the pre-pandemic (2017–2019) and pandemic (2020–2021) study periods, along with demographic data such as gender, age, marital status, educational level, place of origin, among others, and considered specific cardiovascular diseases as the primary cause of death.

The National Death Information System (SINADEF), a software application of the Ministry of Health of Peru responsible for entering and recording deceased individual data, generating death certificates, and compiling death statistics (19), served as the instrument to determine records of deaths due to cardiovascular diseases.

### 2.4 Data gathering, processing, and analysis

Subjects who passed away across all of Peru from January 1, 2017, to December 2022, as available in SINADEF, were considered. The data extracted from the system were recoded using MS-Excel (Microsoft, Redmond, US) for Windows. From the total deaths for each year, those with cardiovascular diseases as the primary cause of death were selected and included in a data matrix. The data were analyzed using SPSS v24.0 (IBM, Armonk, US), employing descriptive statistics to estimate frequencies. The paired *T*-test was used to determine differences in death cases between the pre-pandemic and pandemic periods, considering a significance threshold of *p* < 0.05.

For georeferencing analysis, ArcGIS v10.3 (Esri, Redlands, US) was employed. Initially, six data frames were created and the continental and provincial maps of Peru were loaded, based on data provided by the National Geographic Institute, at a scale of 1:27,500,000. Using these, an MS-Excel table was created with five sheets for each of the cardiovascular disease-related deaths (e.g., stroke), and an additional sheet with cumulative global information for the 2017–2021 period. The “Join Data” tool was used to incorporate the death information for each year.

## 3 Results

**Figure 1:**
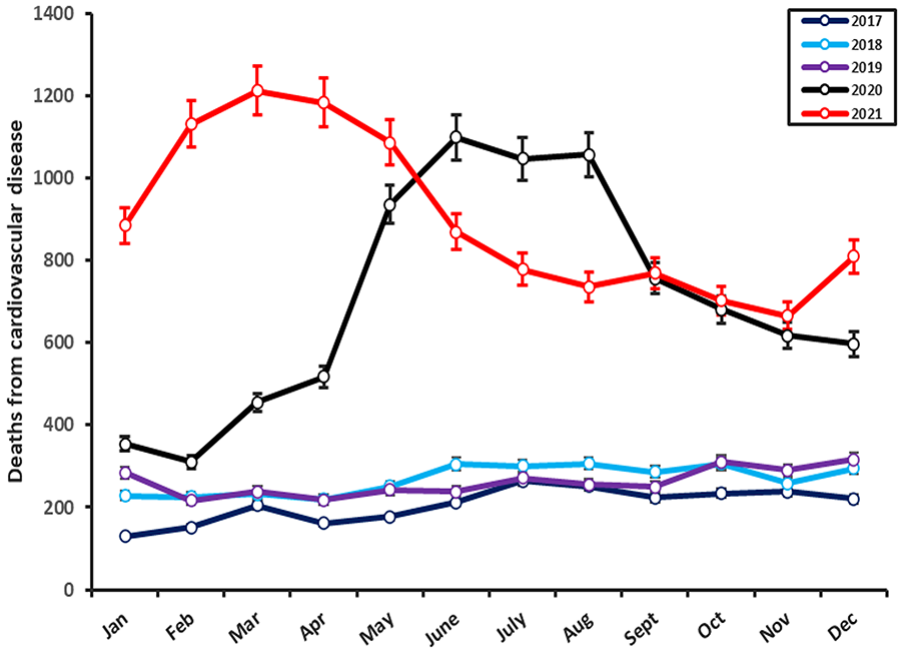
Distribution of deaths from cardiovascular disease in Peru between 2017 and 2022. An increase in the number of deaths (red and black lines) is observed since the start of the response to COVID-19. Both pandemic years show an increase during the waves of COVID-19 infections.

During the analysis period a total of 28,197 deaths were recorded. The pre-pandemic period reported 2,472 (8.8%), 3,215 (11.4%), and 3,134 (11.1%) deaths in 2017, 2018, and 2019, respectively. Meanwhile, during the pandemic, 8,546 (30.3%) and 10,830 (68.7%) deaths were registered in 2020 and 2021, respectively (Supplementary Table S1). A significant increase in cardiovascular mortality cases was observed during the pandemic compared to the pre-pandemic period (19,376 vs. 8,821 deaths, respectively, *p* < 0.001). Figure 1 shows the annual frequency distribution of total CVD deaths, considering the years of COVID-19 lockdowns (2020 and 2021).

In 2017, AMI (1413/2,472) and HF (870/2,472) were the main causes of mortality. These conditions predominantly impacted individuals aged over 60, resulting in 1,154 and 714 deaths in this age group for AMI and HF, respectively. While AMI claimed more male lives (786 deaths), HF affected a higher number of females (461 deaths). In 2018, AMI (2,013/3,215) and HF (970/3,215) were the primary causes of deaths, affecting the elderly primarily (2,636 deaths), and males and females (492 deaths), respectively. In 2019, AMI (1,771/3,134) and HF (615/3,134) remained at the forefront as the leading causes of mortality.The trend persisted with individuals over 60 years old bearing the highest burden, accounting for 2,579 deaths. Notably, AMI continued to affect more males (1,219 deaths), while HF had a greater impact on females (380 deaths).

In 2020, AMI (5,774/8,546) and HF (2,226/8,546) stood out as the primary culprits of mortality, primarily affecting those over 60 years of age (7,328 deaths). AMI claimed more male lives, with 3,158 deaths, while HF proved to be more fatal among females, resulting in 1,130 deaths. In 2021, the trend persisted with AMI (6,805/10,830) and HF (3,382/10,830) maintaining their positions as the primary causes of mortality. Once again, individuals over 60 bore the brunt, accounting for 7,328 deaths. In this year, AMI continued to affect more males, with 3,158 deaths, while HF led to more fatalities among females, reaching 9,169 deaths.

**Figure 2:**
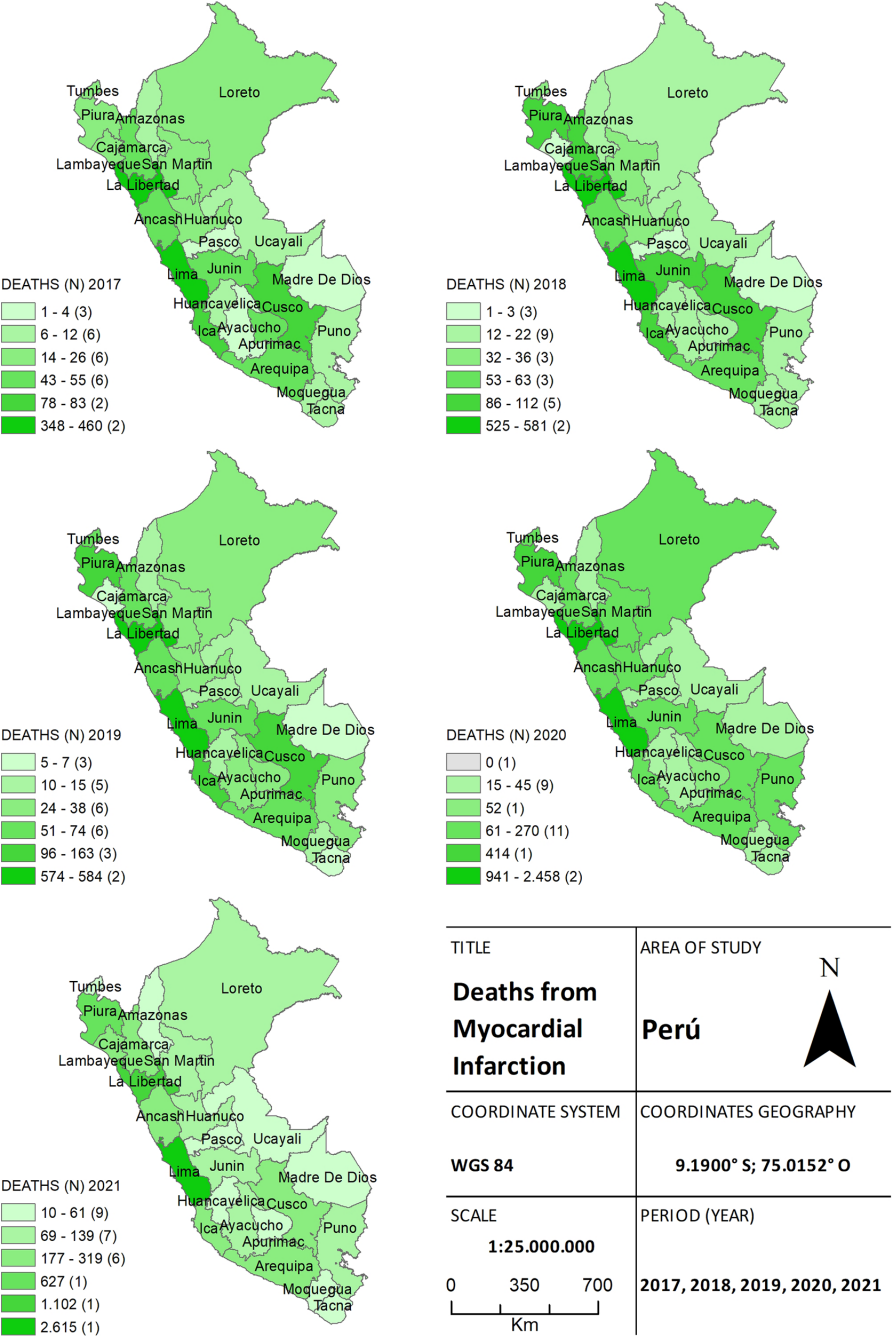
The choropleth map of heart attack, for both the pre-pandemic period (2017–2019) and during the health crisis (2020–2021), provides a detailed view of the geographical distribution of deaths by province. The color gradient on the map reflects the mortality density, aiding in distinguishing areas of higher and lower incidence.

Throughout the study period, a total of 18,152 deaths were attributed to AMI. Of these, 1,413 (7.8%) deaths were reported in 2017, 2013 (11.1%) in 2018, and 2,147 (11.8%) in 2019. During the pandemic, 5,774 (31.8%) and 6,805 (37.42%) deaths were registered in 2020 and 2021, respectively. Significant differences in the quantity of deaths were observed between pre-pandemic years (2017 to 2019) and pandemic years (2020 and 2021) (*p* = 0.017). Georeferencing analysis, as shown in Figure 2, enabled the identification of cases concentrated in coastal cities, including the capital Lima and La Libertad, with a reduction in deaths in certain cities in the highlands (e.g., Cuzco).

**Figure 3:**
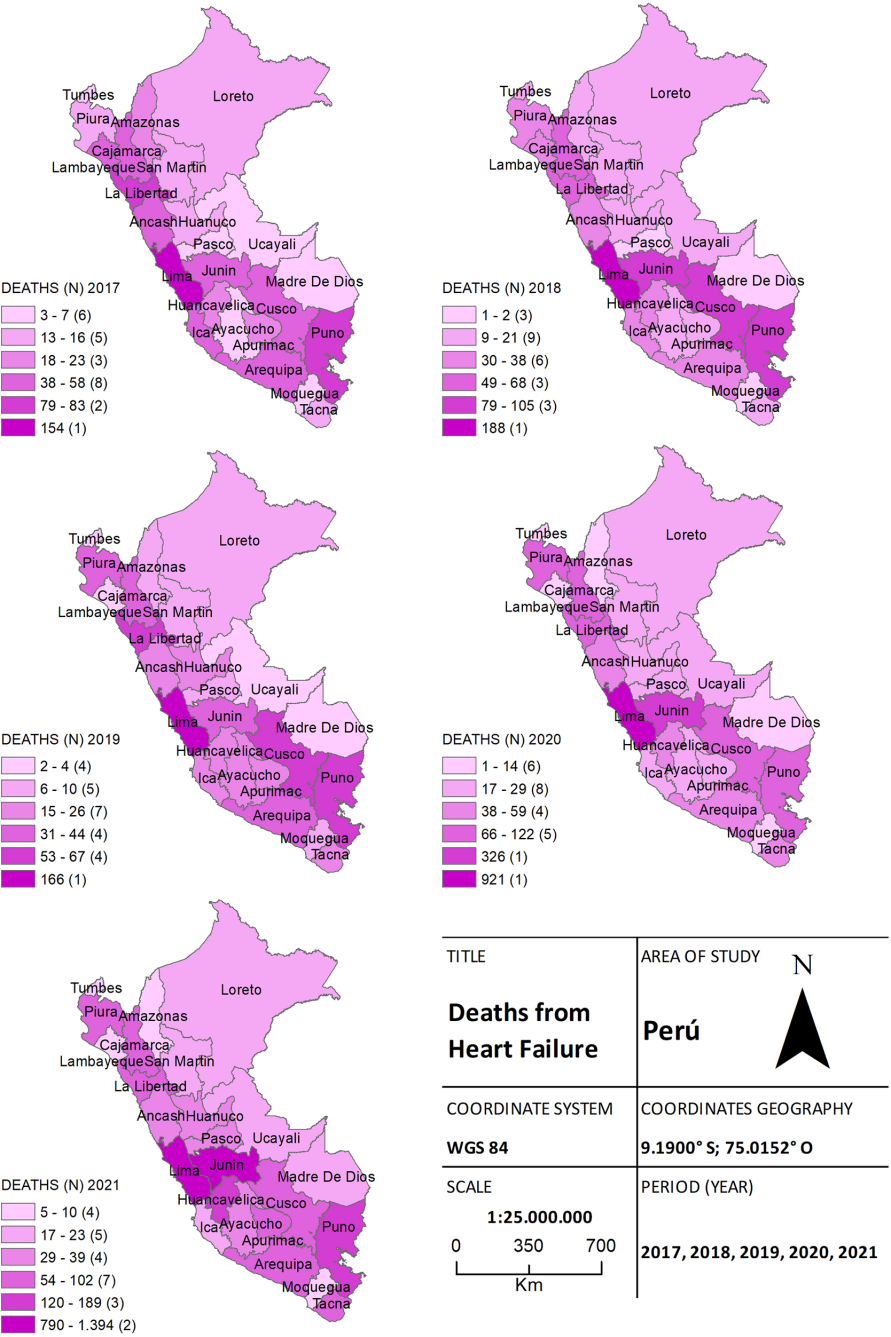
The choropleth map of heart failure, for both the pre-pandemic period (2017–2019) and during the health crisis (2020–2021), provides a detailed view of the geographical distribution of deaths by province. The color gradient on the map reflects the mortality density, aiding in distinguishing areas of higher and lower incidence.

A total of 8,212 deaths due to HF were also recorded. Of these, 870 (10.7%) deaths were reported in 2017, 970 (11.8%) in 2018, and 744 (9.1%) in 2019. During the pandemic, 2,246 (27.3%) and 3,382 (41.2%) deaths were registered in 2020 and 2021, respectively. Significant differences in the frequency of deaths were observed for each province during the pre-pandemic and pandemic years (*p* = 0.002). Georeferencing analysis demonstrated that HF deaths during the pandemic were concentrated in the provinces of Lima and Callao on the coast, and Junin and Puno in the highlands. Pre-pandemic periods showed a heterogeneous distribution (Figure 3).

**Figure 4:**
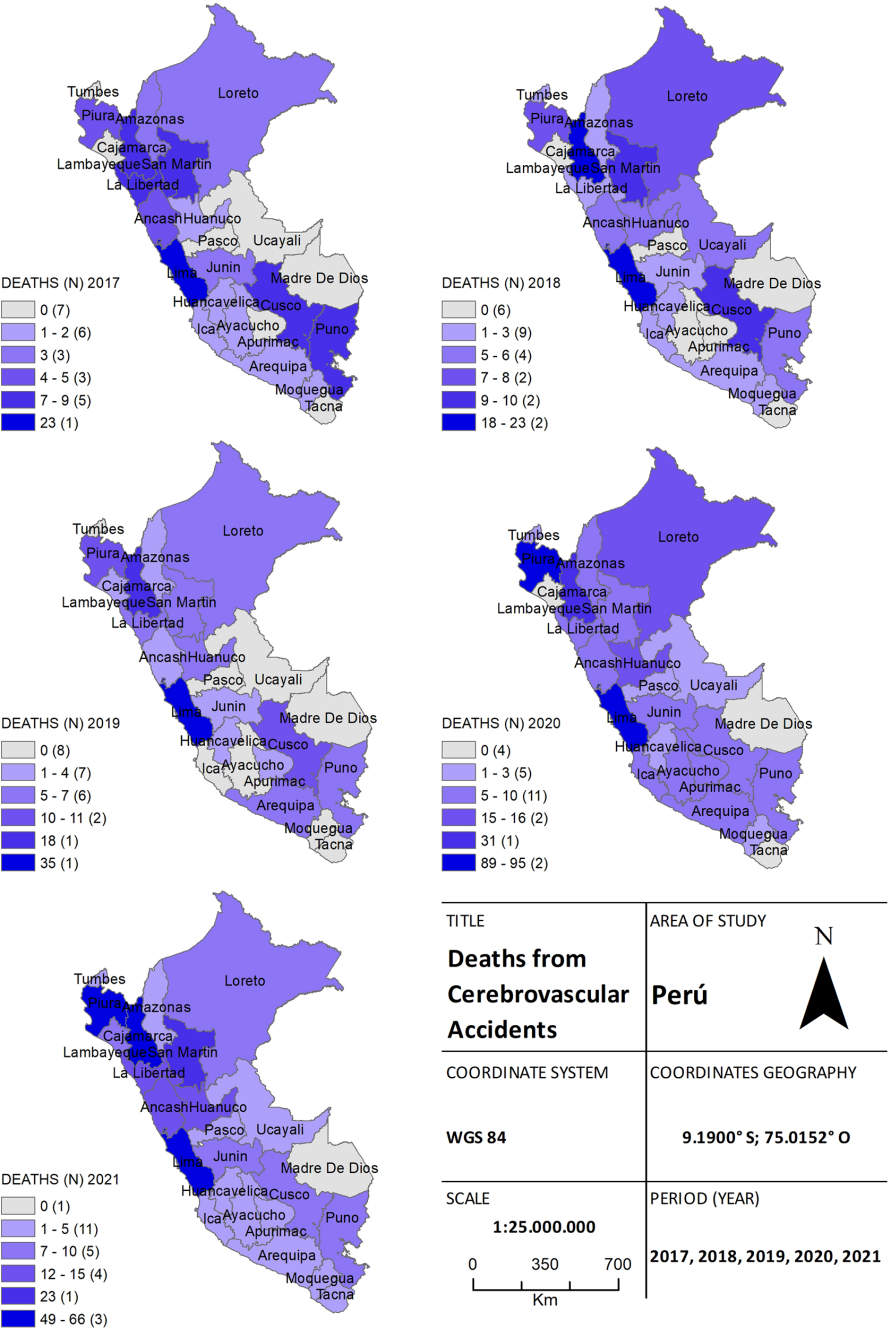
The choropleth map of stroke, for both the pre-pandemic period (2017–2019) and during the health crisis (2020–2021), provides a detailed view of the geographical distribution of deaths by province. The color gradient on the map reflects the mortality density, aiding in distinguishing areas of higher and lower incidence.

In Figure 4, we can see the geographical distributions of the 1,002 stroke-related deaths in both study periods. Of these, 94 (9.4%) deaths were reported in 2017, 117 (11.7%) in 2018, and 125 (12.5%) in 2019. During the pandemic, 341 (34%) and 325 (32.4%) deaths were registered in 2020 and 2021, respectively. Focusing on cities with high stroke mortality during the pandemic, we can notice that the provinces of Piura, Ancash, Lima, and Callao experienced significant increases (*p* = 0.011). While cases worsened in the Highlands of Peru in the province of Cajamarca and Huánuco, the rest of the provinces did not exhibit significant increases (*p* = 0.056).

**Figure 5:**
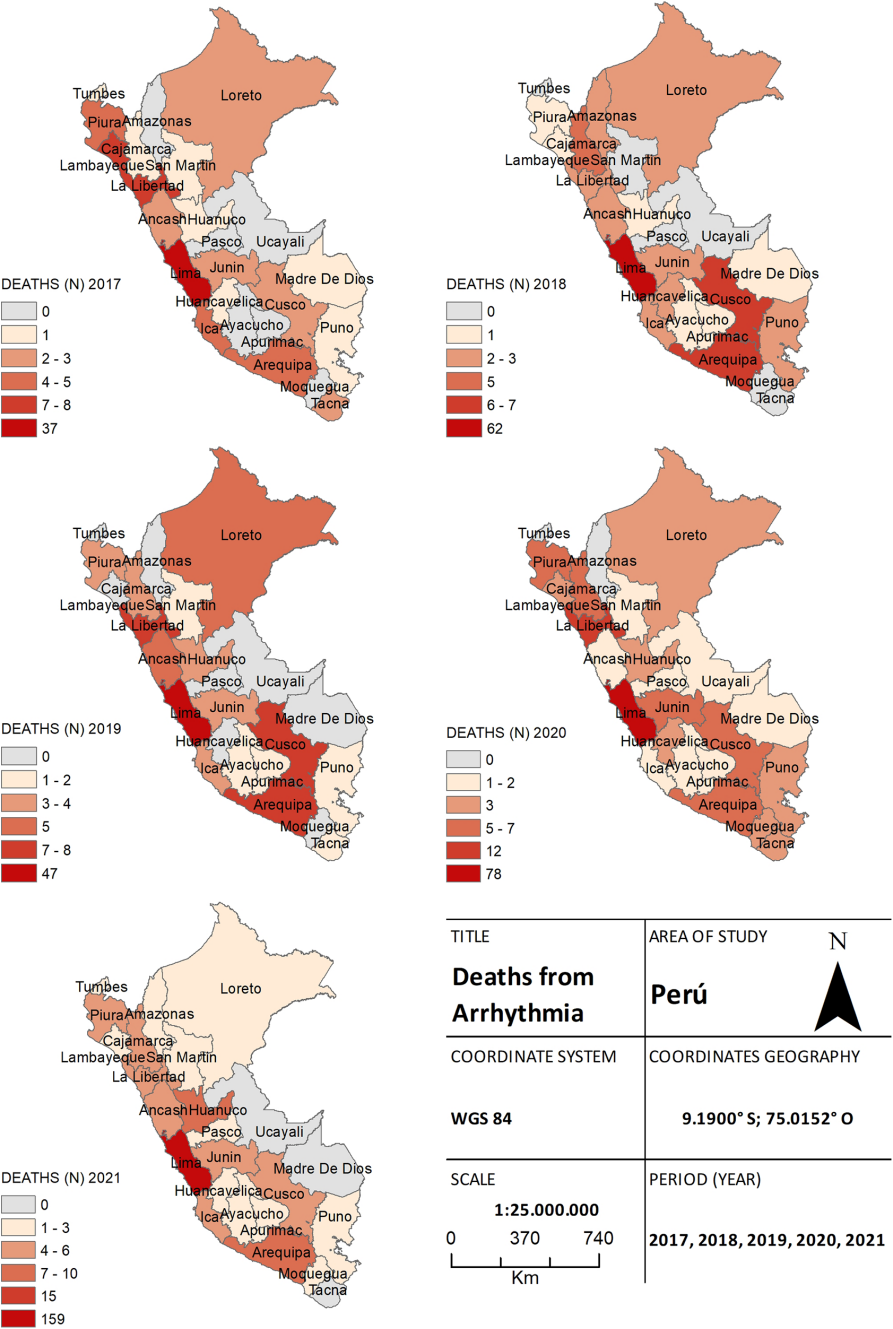
The choropleth map of arrhythmia, for both the pre-pandemic period (2017–2019) and during the health crisis (2020–2021), provides a detailed view of the geographical distribution of deaths by province. The color gradient on the map reflects the mortality density, aiding in distinguishing areas of higher and lower incidence.

A total of 706 deaths due to arrhythmias were also recorded. Among these, 88 (12.5%) deaths were reported in 2017, 108 (15.3%) in 2018, and 110 (15.6%) in 2019. During the pandemic, 157 (22.2%) deaths were recorded in 2020, and 243 (34.4%) in 2021. No significant differences were observed in arrhythmia cases between both periods, as the frequency remained consistent among the provinces of Lima and the southern regions of Peru (Figure 5).

**Figure 6:**
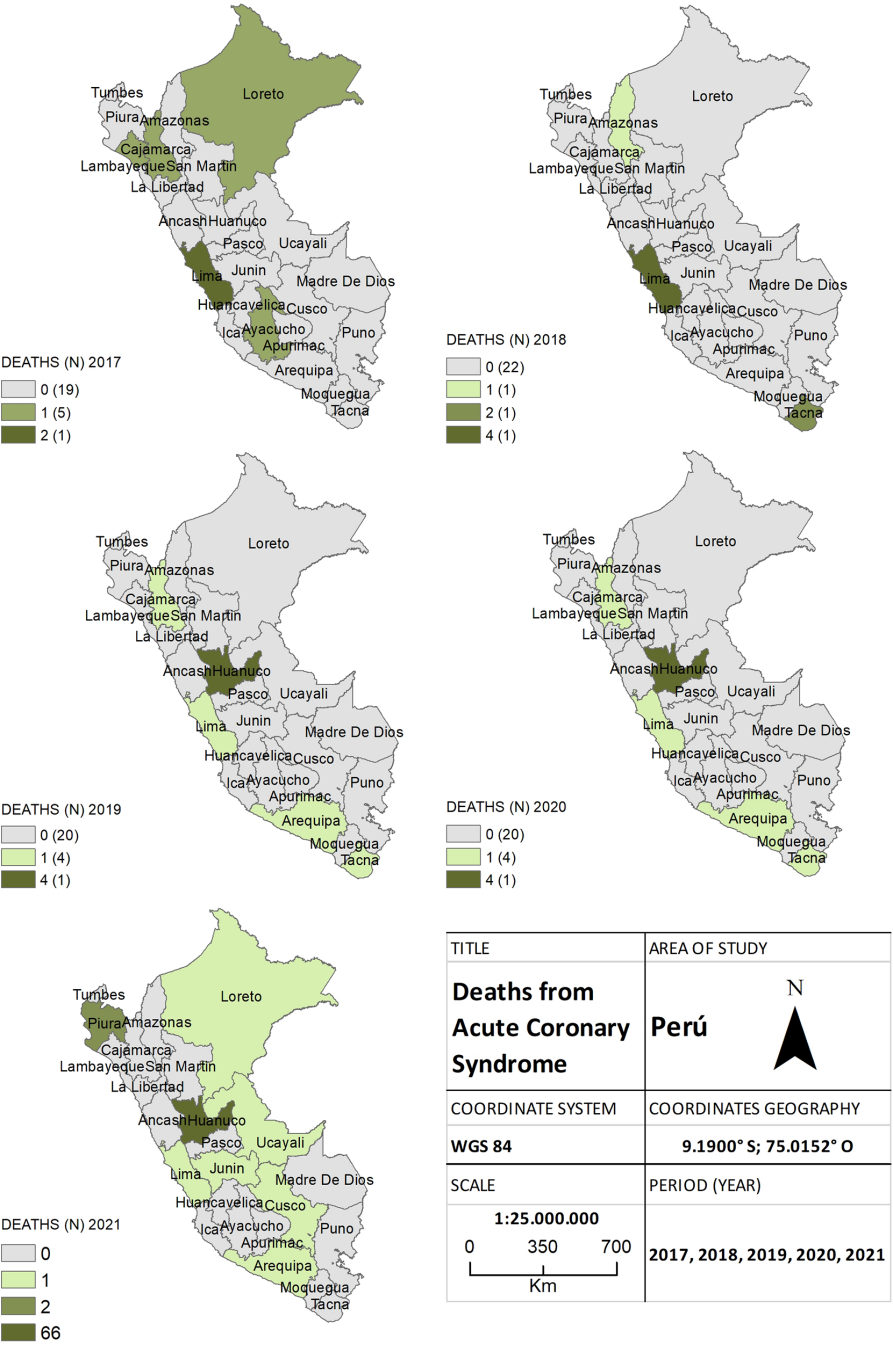
The choropleth map of acute coronary syndrome, for both the pre-pandemic period (2017–2019) and during the health crisis (2020–2021), provides a detailed view of the geographical distribution of deaths by province. The color gradient on the map reflects the mortality density, aiding in distinguishing areas of higher and lower incidence.

Finally, a total of 125 cases of acute coronary syndrome were recorded. Among these cases, 22 (17.6%) were reported between 2017 and 2019, whereas during the pandemic, 28 (22.4%) cases were reported in 2020, and 75 (60%) in 2021 (Figure 6). Significant differences were found in the frequency of deaths between both periods, and it was identified that the province of Huánuco and several provinces within the jungle region experienced an increase in cases (*p* = 0.005).

## 4 Discussion

This geoepidemiological study, conducted in a country with high COVID-19 mortality rates, brings to light and underscores territorial heterogeneity in mortality trends due to cardiovascular diseases (CVD) before and during the pandemic. Our findings reveal a noteworthy increase in the number of deaths due to CVD during the pandemic years in comparison to the pre-pandemic era. This suggests a potential impact of the health crisis on the cardiovascular health of the population. Notably, most cases of myocardial infarction and HF during the COVID-19 pandemic were concentrated within the coastal provinces of Peru. However, some CVD-related deaths also showed an increase in the highland's regions.

### 4.1 Strengths

To the best of our knowledge, this study has, for the first time, demonstrated the geospatial distribution of COVID-19 deaths in Peru. While previous reports have analyzed other pandemic outcomes (7, 9, 10, 20, 21), they have not explored shifts in the distribution of CVD-related deaths before and during the pandemic. These results underscore the persistent nature of cardiovascular diseases, particularly AMI and HF, as leading causes of mortality. Another strength of this study lies in the use of official and comprehensive five-year data reported by the SINADEF of the Ministry of Health (19). This minimizes the possibilities of bias and error in the compilation of information on cardiovascular disease-related deaths, thus reinforcing the robustness of the study's conclusions. The spatial and temporal analysis further contributes to the strengths of the study, offering both a visual and spatial comprehension of mortality patterns (22). Additionally, the focus on specific periods (pre-pandemic and pandemic) facilitates the identification of temporal trends and the assessment of pandemic impact on outcomes.

### 4.2 Main findings

Primarily, a significant increase in total deaths due to CVD was observed during the pandemic period, with 19,376 deaths recorded compared to 8,821 deaths in the pre-pandemic period. Previous reports (6, 23–29) support our findings and indicate that CVD is a primary mortality factor in COVID-19 patients. These outcomes imply an association between the pandemic and increased CVD-related deaths, potentially attributed to factors such as limited access to medical care (6), treatment postponements (7), pre-existing CVD (8, 26), and the impact of pandemic-related stress.

Globally, COVID-19 has caused an excess of CVD-related deaths, significantly affecting low- and middle-income countries (6, 8, 24, 25, 27, 30). Individuals hospitalized due to the virus have demonstrated a higher risk of CVD-related deaths (6, 28). However, it is essential to note that CVD rates within hospitalizations exhibited a relative decrease, suggesting a considerable number of deaths occurred outside of hospitals, such as in homes or clinics (24). For instance, in socially vulnerable cities like Belo Horizonte in Brazil, higher rates of non-hospital deaths were observed (29). This phenomenon can be attributed to significant shifts in healthcare delivery induced by the COVID-19 pandemic. During the peak of the disease, healthcare systems often became overwhelmed, leading to situations where individuals lacked access to proper medical care and opted to remain at home or passed away while going to healthcare facilities. In the case of Peru, a comprehensive analysis of excess CVD-related mortality considering both the place of death and the type and level of medical care provided is crucial. This will provide a better understanding of the unique impact of the pandemic on the country and support the formulation of effective strategies to address the interplay between COVID-19 and cardiovascular diseases.

The detailed analysis of CVD-related causes of death during the studied years revealed intriguing patterns. The most frequent CVDs were heart attack and HF, resulting in 12,579 and 5,628 deaths during the pandemic, respectively. These figures are more than double those recorded in the three-year period prior to the outbreak. These results align with various prior investigations, reinforcing the position of CVDs as primary mortality causes. In the United States, multiple studies have identified AMI as the main contributor to CVD-related deaths (23, 26, 31), while the United Kingdom has identified HF and stroke as the leading causes (28). It is possible that the cluster of pre-existing conditions including CVD, hypertension, diabetes, and obesity (26, 30–33) play a determinative role in the observed increase in mortality rates. Furthermore, the unique nature of COVID-19, as manifested through distinct epidemiological and clinical characteristics in each outbreak (34), could also contribute to increased mortality rates. Comprehensive understanding of the intricate interplay among these variables sheds light on the dynamics and driving forces behind the surge in CVD-related mortality during the COVID-19 pandemic.

An interesting finding in our study was the geographic distribution of CVD-related deaths during the pandemic. A significant increase in deaths due to AMI was observed in specific provinces, while deaths due to HF were concentrated in other geographic areas. Upon analyzing different regions, it was shown that coastal provinces, characterized by high urbanization, population density, and high contagion rates, experienced pronounced increases in CVD-related deaths. For instance, the United States has witnessed higher mortality rates in New York compared to other states like Massachusetts or Louisiana (31). These disparities could also be influenced by socio-economic factors, access to medical care, suboptimal health response, and other contextual variables specific to each region (35, 36).

The interaction among these elements suggests a complex interconnectedness that amplifies the pandemic's effects on cardiovascular mortality. Geographical and socio-economic factors, coupled with healthcare disparities and health responses, contribute to this substantial variation in mortality patterns. Understanding these dynamics is imperative for designing precise and effective public health approaches aimed at addressing the specific challenges faced by each region, thus mitigating adverse impacts on cardiovascular health during the pandemic and similar future events.

Finally, this study holds clinical significance, as even in Latin America, records of COVID-19-related cardiovascular deaths are limited. Few reports have indicated an increase in CVD deaths in Ecuador and Mexico by 48.6% and 34.9%, respectively (30). Given the varied pandemic responses (2) and the fluctuating rates of infection and mortality based on implemented control measures (37), conducting national-level studies is imperative to comprehensively address risk factors (12), understand excess CVD-related mortality, and evaluate changes in mortality in relation to the initiation of COVID-19 vaccination efforts in the region (11).

### 4.3 Limitations

First, georeferencing and geographic analysis were based on aggregated provincial-level data, potentially overlooking local-level differences (9). Populations in Latin America, particularly in rural and peri-urban areas, have experienced decreased wellbeing as COVID-19 deaths have increased (38). In this regard, it is essential to conduct analyses based on income and healthcare accessibility. Second, there might be ethnic and gender disparities that need deeper exploration, as differences have been observed between pandemic waves (27). Our study primarily focused on developing a geospatial analysis of CVD deaths and did not evaluate factors such as hypertension, obesity, and diabetes, which can significantly impact death rates (23, 25, 31, 32). Additionally, although an association between the pandemic and increased CVD deaths was identified, a definitive causal relationship cannot be established due to the observational nature of the study. Finally, issues have been reported regarding the misclassification of COVID-19 deaths and the categorization of CVD as a primary cause (30), potentially introducing biases in SINADEF data and affecting result interpretation. Despite these acknowledged limitations, this study offers a comprehensive view of CVD death patterns in the Peruvian population over the past five years, with a specific focus on the influence of the COVID-19 pandemic.

## 5 Conclusions

Our results underscore the significance of addressing cardiovascular diseases as a public health concern, particularly during crises such as the COVID-19 pandemic. CVD deaths, particularly AMI and HF, have experienced a significant increase during the pandemic, revealing a distribution pattern among Peruvian provinces. Identifying specific mortality patterns for CVD, along with their geographical variability, can inform targeted preventive and healthcare strategies. Future research should continue exploring the underlying factors contributing to the rise in CVD deaths during the pandemic and develop effective interventions to mitigate this im-pact.

## Data Availability

Publicly available datasets were analyzed in this study. This data can be found here: https://www.minsa.gob.pe/defunciones/.
