# Supplementary material for: Geospatial analysis of cardiovascular mortality before and during the COVID-19 pandemic in Peru: analysis of the national death registry to support emergency management in Peru
Source: Front Cardiovasc Med. 2024 Jun 3;11:1316192. doi: 10.3389/fcvm.2024.1316192 (PMC11180746; doi:10.3389/fcvm.2024.1316192)
Supplement: Supplementary file 1 [file Table1.docx]

**Suppl. 1.** Distribution of deaths according to CVD during the pre-endemic and pandemic years due to COVID-19. Data is shown by region and global deaths

| **Global** | | | | | | | | | | | | |
| --- | --- | --- | --- | --- | --- | --- | --- | --- | --- | --- | --- | --- |
| Region (province) | Stroke | | Arrhythmia | | Heart attack | | HF | | IMA | | Total | |
| Amazonas | 17 | (1.7) | 4 | (0.6) | 86 | (0.5) | 63 | (0.8) | 1 | (0.8) | 171 | (0.6) |
| Ancash | 33 | (3.3) | 16 | (2.3) | 674 | (3.8) | 182 | (2.3) | 1 | (0.8) | 906 | (3.3) |
| Apurimac | 12 | (1.2) | 6 | (0.9) | 198 | (1.1) | 138 | (1.7) | 0 | (0.0) | 354 | (1.3) |
| Arequipa | 24 | (2.4) | 35 | (5.0) | 620 | (3.5) | 227 | (2.8) | 2 | (1.6) | 908 | (3.3) |
| Ayacucho | 11 | (1.1) | 5 | (0.7) | 168 | (0.9) | 106 | (1.3) | 1 | (0.8) | 291 | (1.0) |
| Cajamarca | 128 | (12.9) | 19 | (2.7) | 654 | (3.6) | 309 | (3.9) | 2 | (1.6) | 1112 | (4.0) |
| Callao | 22 | (2.2) | 26 | (3.7) | 294 | (1.6) | 363 | (4.5) | 2 | (1.6) | 707 | (2.5) |
| Cusco | 42 | (4.2) | 29 | (4.1) | 636 | (3.5) | 362 | (4.5) | 1 | (0.8) | 1070 | (3.9) |
| Huancavelica | 12 | (1.2) | 8 | (1.1) | 112 | (0.6) | 242 | (3.0) | 0 | (0.0) | 374 | (1.3) |
| Huánuco | 44 | (4.4) | 16 | (2.3) | 248 | (1.4) | 112 | (1.4) | 87 | (69.6) | 507 | (1.8) |
| Ica | 13 | (1.3) | 16 | (2.3) | 889 | (5.0) | 145 | (1.8) | 0 | (0.0) | 1063 | (3.8) |
| Junín | 22 | (2.2) | 17 | (2.4) | 410 | (2.3) | 1285 | (16.1) | 1 | (0.8) | 1735 | (6.2) |
| La Libertad | 36 | (3.6) | 33 | (4.7) | 3612 | (20.1) | 332 | (4.1) | 0 | (0.0) | 4013 | (14.4) |
| Lambayeque | 9 | (0.9) | 13 | (1.9) | 221 | (1.2) | 86 | (1.1) | 1 | (0.8) | 330 | (1.2) |
| Lima | 237 | (23.9) | 383 | (54.8) | 6576 | (36.6) | 2823 | (35.3) | 12 | (9.6) | 10031 | (36.1) |
| Loreto | 40 | (4.0) | 14 | (2.0) | 195 | (1.1) | 71 | (0.9) | 2 | (1.6) | 322 | (1.2) |
| Madre De Dios | 0 | (0.0) | 3 | (0.4) | 37 | (0.2) | 37 | (0.5) | 0 | (0.0) | 77 | (0.3) |
| Moquegua | 5 | (0.5) | 5 | (0.7) | 103 | (0.6) | 33 | (0.4) | 0 | (0.0) | 146 | (0.5) |
| Pasco | 3 | (0.3) | 4 | (0.6) | 67 | (0.4) | 61 | (0.8) | 0 | (0.0) | 135 | (0.5) |
| Piura | 178 | (17.9) | 19 | (2.7) | 1260 | (7.0) | 231 | (2.9) | 2 | (1.6) | 1690 | (6.1) |
| Puno | 34 | (3.4) | 9 | (1.3) | 237 | (1.3) | 463 | (5.8) | 0 | (0.0) | 743 | (2.7) |
| San Martín | 54 | (5.4) | 8 | (1.1) | 284 | (1.6) | 84 | (1.0) | 0 | (0.0) | 430 | (1.5) |
| Tacna | 2 | (0.2) | 6 | (0.9) | 103 | (0.6) | 161 | (2.0) | 9 | (7.2) | 281 | (1.0) |
| Tumbes | 4 | (0.4) | 3 | (0.4) | 204 | (1.1) | 31 | (0.4) | 0 | (0.0) | 242 | (0.9) |
| Ucayali | 10 | (1.0) | 2 | (0.3) | 70 | (0.4) | 58 | (0.7) | 1 | (0.8) | 141 | (0.5) |
| TOTAL | 992 | (100) | 699 | (100) | 17958 | (100) | 8005 | (100) | 125 | (100) | 27779 | (100) |
